# Picroside II attenuates hyperhomocysteinemia‐induced endothelial injury by reducing inflammation, oxidative stress and cell apoptosis

**DOI:** 10.1111/jcmm.13949

**Published:** 2018-11-05

**Authors:** Yunkai Wang, Yajun Hong, Chunyu Zhang, Yunli Shen, Ye Shen Pan, Rui Zhen Chen, Qi Zhang, Yi Han Chen

**Affiliations:** ^1^ Department of Cardiology Shanghai East Hospital Tongji University School of Medicine Shanghai China; ^2^ Department of Radiology Fudan University Shanghai Cancer Center Shanghai China; ^3^ Department of Cardiology Zhongshan Hospital Fudan University Shanghai China

**Keywords:** atherosclerosis, hyperhomocysteinemia, LOX‐1, oxidative stress, Picroside II, SIRT1

## Abstract

Picroside II (P‐II), one of the main active components of scrophularia extract, which have anti‐oxidative, anti‐inflammatory effects, but its effect on hyperhomocysteinemia (HHcy) induced endothelial injury remains to be determined. Here, we test whether P‐II protects HHcy‐induced endothelial dysfunction against oxidative stress, inflammation and cell apoptosis. In vitro study using HUVECs, and in hyperhomocysteinemia mouse models, we found that HHcy decreased endothelial SIRT1 expression and increased LOX‐1 expression, subsequently causing reactive oxygen species generation, up‐regulation of NADPH oxidase activity and NF‐κB activation, thereby promoting pro‐inflammatory response and cell apoptosis. Blockade of Sirt1 with Ex527 or siRNASIRT1 increased LOX‐1 expression, whereas overexpression of SIRT1 decreased LOX‐1 expression markedly. P‐II treatment significantly increased SIRT1 expression and reduced LOX‐1 expression, and protected against endothelial cells from Hcy‐induced oxidative injury, inflammation and apoptosis. However, blockade of SIRT1 or overexpression of LOX‐1 attenuated the therapeutic effects of P‐II. In conclusion, our results suggest that P‐II prevents the Hcy induced endothelial damage probably through regulating the SIRT1/LOX‐1 signaling pathway.

## 1 INTRODUCTION

During the past decades, the convergence of clinical and basic evidence has demonstrated a fundamental role for endothelial dysfunction in pathogenesis and development of atherosclerotic diseases.^1^ Homocysteine (Hcy) is derived from sulfur‐containing and non‐proteinogenic amino acid, and during the metabolism of methionine, it is formed in trace amount. Hyperhomocysteinemia (HHcy) can induces vascular endothelial injury, and has been determined to be one of the main risk factors for cardiovascular diseases,^2^, ^3^ but its exact mechanism remains unclear.^4^ Lectin‐like oxLDL receptor‐1 (LOX‐1) is an essential receptor for oxide low‐density lipoprotein (oxLDL),^5^ and plays a vital role in atherosclerotic process.^6^ Much studies have shown that HHcy up‐regulated human mononuclear and endothelial cells LOX‐1 expression,^7^, ^8^ which prompts reactive oxygen species (ROS) formation,^8^, ^9^ induces pro‐apoptotic signal transduction and nitric oxide (NO) catabolism, consequently causing oxidative injury and the endothelial cells death.^10^, ^11^

Picrorrhiza scrophulariiflora belongs to the plant family, scrophularia. The roots of this plant are of benefit, and as a traditional medicine, it is used for a number of conditions.^12^, ^13^ Picroside II (P‐II), one of the main active components of scrophularia extract, has shown its anti‐oxidative, anti‐inflammatory effects.^14^, ^15^, ^16^ However, whether P‐II could inhibit HHcy‐induced endothelial injuries has not been studied. In this study, we assess the protective effect of P‐II on endothelial injury induced by HHcy and to determine its possible regulatory mechanisms.

## 2 MATERIALS AND METHODS

### 2.1 Drugs and reagents

Picroside II was purchased from Shanghai Standard Biotech (Shanghai, China) at a purity of 98%. It is stocked in dimethylsulfoxide (DMSO) solution and stored at −30°C. Hyperhomocysteinemia was purchased from Sigma (St. Louis, MO, USA). TRizol was obtained from Invitrogen (California, USA), the LOX‐1, SIRT1 and Caspase‐3 antibody were purchased from Santa Cruz Biotechnology Inc. (California, USA).

Hcy(D090821), DCFH‐DA(D6883), DMSO(D5879), Hepes, SOD, MDA and NADPH oxidase activity kits were got from Sigma‐Aldrich (USA). IL‐6, IL ‐8, CXCL15 and TNF‐α ELISA test kit were purchased from Shanghai Hong LiBiotechnology Company.

### 2.2 Animals

In this study, a total of 50 C57BL mice were randomly divided into five experimental groups (10 in each group): sham control (PBS‐treated), HHcy‐induced group, HHcy+P‐II treatment group (low dose:10 mg/kg), and HHcy+P‐II treatment group (high dose: 60 mg/kg) group, HHcy +P‐II treatment group (high dose: 60 mg/kg) + EX527 group [1.2 mM EX527 diluted in phosphate‐buffered saline (5 μL) was administered intravenously at 2 hours before P‐II treatment]. HHcy model was established by feeding C57BL mice with L‐methionine (w/w)‐rich diet for 12 weeks, and the Hcy levels in the serum were tested .After the 3‐month methionine ‐rich diet, mice were administered with P‐II (low dose:10 mg/kg, or high dose: 60 mg/kg) or PBS orally for another 8 weeks every day.

### 2.3 Cell culture

The cell study protocol was approved by the Research Ethics Committee of Shanghai East Hospital, Tongji University School of Medicine. After receiving written consent from the parents, fresh human umbilical cords from normal full‐term neonates shortly after birth were obtained and suspended in Hanks’ balanced salt solution (HBSS; GIBCO) at 4°C. Human umbilical vein endothelial cells (HUVECs) were cultured as previous experiment 17. Briefly, after collagenase type I digestion, HUVECs were got from human umbilical veins, next it is cultured in medium 199 at 37°C in 5% CO_2_ on 0.1% gelatin‐coated culture flasks. The medium 199 contains 20% fetal calf serum, penicillin (100 U/mL), streptomycin (100 U/mL) and heparin (50 U/mL), and supplemented with 2 mM L‐glutamine, 1 mM sodium pyruvate and 5 ng/mL endothelial cell growth factor. When endothelial cells morphology appears “cobblestone” mosaic appearance and the presence of von Willebrand factor, it is identified. Experiments were repeated in HUVECs from passage 2 to 7, with no differences observed between passages.

For experiments, HUVECs were divided into six groups randomly: a normal control group, a Hcy group, three P‐II groups. Cells in the control group were incubated under the normal growth conditions. The HUVECs in the Hcy group were incubated with medium containing 100 μmol Hcy for 24 hours. In the P‐II groups, the cells were preincubated with different concentrations (50 μg/mL, 100 μg/mL, 200 μg/mL) of P‐II for 24 h, and next it is incubated with 100 μmol Hcy for another 24 hours.

### 2.4 Cell viability

CCK‐8 assay were used for cell viability determination. Briefly, HUVECs were first inoculated in 96‐well culture plates, and adjusted to the concentration of 4 × 105 cells/mL (200 μL/well). With 12 hours incubation, supernatant was abandoned, and then the cells were treated with different concentrations of P‐II (50 μg/mL, 100 μg/mL, 200 μg/mL) for 48 hours. At last, in the all of the groups, CCK‐8 (10 μL/well) were added and incubated for the next 3 hours. On a microplate reader, the optical density was measured at 450 nm.

### 2.5 Overexpression or knockdown of LOX‐1 and SIRT1

The pCMV6‐XL5‐LOX‐1 and SIRT1 plasmids, from Origene Technologies (Rockville, MD, USA), were constructed with full‐length human LOX‐1 cDNA OR SIRT1 and transfected into HUVECs with a FuGene 6 transfection reagent (Roche Diagnostics, Mannheim, Germany).^18^ Empty vectors were transfected as controls. After 48 hours transfection, HUVECs were incubated with P‐II and HHcy. Then, cells were collected for analysis.

For siRNA experiments, HUVECs were transfected with LOX‐1 siRNA or SIRT1 siRNA (Santa Cruz Biotechnology). Briefly, HUVECs were cultured in antibiotic‐free Dulbecco's modified Eagle's medium at 37°C for 24 hours, then the siRNA duplex solution was added. Cells were subjected to each experiment 24 hours after transfection.

### 2.6 RNA preparation and RT‐PCR analysis

According to the manufacturer's protocol, we extracted the total RNA from HUVECs or mouse aortic rings with Trizol reagent. Next, using M‐MLV reverse transcriptase, we reversely transcribed RNA into cDNA. The resultant cDNA was detected by Real‐time PCR.^17^

### 2.7 Western blotting

First, we lysed total protein from human umbilical vein cells or mouse aortic rings with modified RIPA lysis buffer on ice for 30 minutes.^17^ Then, the lysate was centrifuged at 14 000 *g* for 15 minutes to precipitate the unsolvable materials. Next, we determined protein concentrations by the Bio‐Rad protein assay kit. Samples were electrophoresed in SDS‐PAGE gels and separated proteins were transferred to a PVDF membrane. The blots were blocked with 5% non‐fat dry milk in Tris‐buffered saline Tween‐20 (TBST) for 1 hour at room temperature and subsequently incubated overnight at 4°C with appropriate primary antibody. After three washes with TBST, the blots were incubated with horseradish peroxidase‐conjugated secondary antibodies in blocking buffer for 1 hour at room temperature. At last, antigen was detected using enhanced chemiluminescence (ECL).

### 2.8 Elisa

According to the manufacturer's instructions, SOD, MDA and chemokines in the supernatants or plasma were determined using ELISA kits. The levels of IL‐6, IL‐8, CXCL15 and TNF‐α were measured by EnSpire Multimode Plate Readers (PerkinElmer, Fremont, CA, USA) at the absorbance at 450 nm.

Plasma levels of Hcy were measured using an Hcy detection kit (enzymatic cycling assay) on cobas c311 automatic biochemical analyser (Roche, Switzerland) .

### 2.9 Assay of intracellular ROS production

10 μM of the fluorescent probe, CMH2DCF‐DA (2′‐7′‐dichlorodihydrofluorescein diacetate; Sigma‐Aldrich, St. Louis, MO, USA), was added into Confluent HUVECs (96‐well plates). 30 minutes later, Fluorescence intensity was measured by using a microplate reader (BioTek Instruments) at excitation 490 nm and emission 540 nm.

### 2.10 Assay of NADPH oxidase activity

HUVECs and mouse aortic rings were harvested respectively. NADPH‐enhanced superoxide (O_2_^−^) release in HUVECs or mouse aortic rings homogenate were calculated using lucigenin‐enhanced chemiluminescence (CL), as previously described.^19^

### 2.11 Cellular MDA levels, SOD and catalase activity measurement

According to Cayman's assay kits instruction (Cayman Chemical, Ann Arbor, MI, USA), the levels of MDA and the activity of SOD and catalase (CAT) in HUVECs homogenate were determined.

### 2.12 Apoptosis assessment

By analysis of DNA fragmentation, apoptosis was examined using flow cytometry.^20^ First, by using an Annexin V‐FITC apoptosis detection kit, HUVECs were washed and double‐stained. As we know, Annexin V can translocates from the internal to the external surface of the plasma membrane for its strong Ca^2+^‐dependent affinity for phosphatidylserine (PS), and can detect apoptosis as a probe. Cells with the loss of membrane integrity will show red staining (propidium iodide, PI) throughout the nucleus, so the early apoptotic cells and the late apoptotic cells or necrotic cells are easily distinguished. At room temperature, samples were incubated in the dark with Annexin V and PI for 15 minutes, and then they were analyzed by a FACS vantage SE flow cytometer quantitatively.

According to the caspase‐3 assay kit (Calbiochem) instructions, the activities of caspase‐3 were determined.^21^ Briefly, we lysed and removed HUVECs from each group culture dishes, then washed twice with PBS, and pelleted by centrifugation. Next, cell pellets were treated with iced lysis buffer for 10 minutes. Then the suspensions were centrifuged for 10 minutes at 10 000 *g*, and the supernatants were transferred into a clear tube. Specific substrate conjugate [acetyl‐Asp‐Glu‐Val‐Asp‐p‐nitroaniline (Ac‐DEVD‐ p‐NA) for caspase‐3], was added into each tube, and the tubes were incubated for 2 hours at 37°C. The substrates were cleaved by caspases to form p‐NA or AFC during incubation. Caspase‐3 activities were calculated in a microtiter plate reader at 405 nm. In this study, assays were performed in triplicate and three independent experiments were performed.

### 2.13 Statistical analysis

Differences between two groups were assessed using two‐tailed *t* tests. Differences between more than two groups were assessed using one‐way analysis of variance (ANOVA). To compare the interaction between two factors, two‐way ANOVA tests were performed. ANOVA, assessed by Bonferroni's post hoc test, was used when comparing more than two groups. All *P* < 0.05 were considered significant.

## 3 RESULTS

### 3.1 Characteristics of control and hyperhomocysteinaemic mice

**Figure 1:**
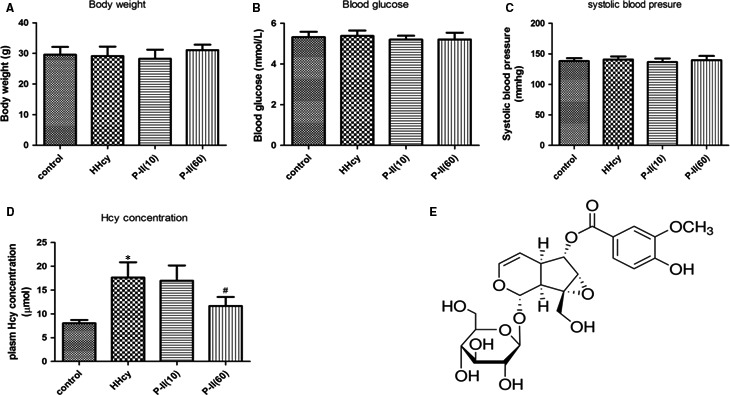
Characteristics of animals and the chemical structure of picroside II. C57BL mice were fed with L‐methionine (w/w) ‐rich diet for 12 weeks, and then Body weight, Systolic blood pressure, blood glucose, Hcy levels in the serum were tested. (A) Body weight. (B) Systolic blood pressure. (C) Blood glucose and (D) Homocysteine levels. (E) The chemical structure of picroside II. *P <* *0.05 as compared to control group mice, ^#^ *P *<* *0.05 as compared to Hcy‐treated mice

There was no significant difference in initial body weight, systolic blood pressure, blood glucose among groups (all *P *>* *0.05) (Figure 1A‐C). Plasma Hcy was increased in animals fed with 1% methionine, while reduced after treatment with P‐II (Figure 1D).

### 3.2 Picroside II attenuated vascular oxidative stress and inflammation in hyperhomocysteinaemic mice

**Figure 2:**
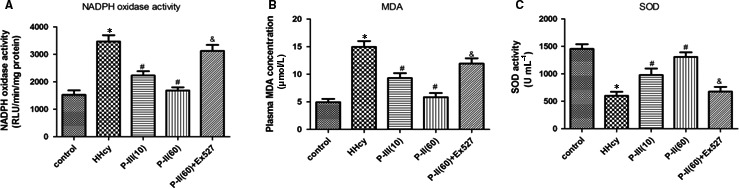
Picroside‐II attenuated vascular oxidative stress in hyperhomocysteinaemic mice. The control mice (Control) had free access to water, and the hyperhomocysteinaemic mice (HHcy) were treated with 2% (m·v‐1) methionine/drinking water for 3 months, then mice were divided into five groups for the next 2 month: the control mice, the HHcy group, the HHcy+P‐II group (treated orally with P‐II low dose:10 mg/kg, or high dose:60 mg/kg) or PBS every day, the HHcy +P‐II treatment (high dose:60 mg/kg)+EX527 group, which 1.2 mM EX527 was prepared in phosphate‐buffered saline for a single intravenous (5 μl) injection at 2 hours before P‐II treatment. (A) The effect of P‐II on the NADPH activation in thoracic arteries. (B‐C) Plasma MDA levels (B) SOD activity (C) were measured. The data are presented as the mean ± SD (n = 10 in each group). *P < 0.05 as compared to control group mice, ^#^ *P* < 0.05 as compared to hyperhomocysteinaemic mice. ^&^ *P* < 0.05 as compared to the HHcy + P‐II treatment (high dose: 60 mg/kg) group mice

As shown in Figure 2A, aortic endothelial NADPH oxidase activity was increased markedly in the HHcy mice group, however after treatment with P‐II, it was decreased (Figure 2A). In addition, the level of plasma MDA in the HHcy mice was higher than that in the control group; however, treatment with P‐II reversed HHcy‐induced MDA increase (Figure 2B). The plasma SOD activity was markedly reduced in the HHcy mice group but was increased significantly after treatment with P‐II (Figure 2C).

**Figure 3:**
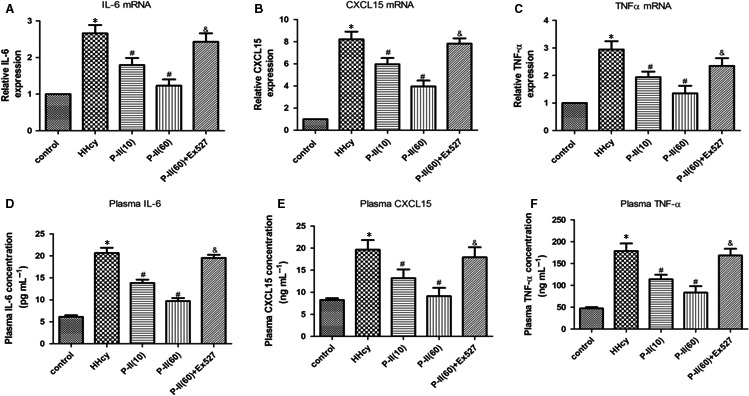
Picroside‐II attenuated vascular inflammation in hyperhomocysteinaemic mice. The control mice (Control) had free access to water, and the hyperhomocysteinaemic mice (HHcy) were treated with 2% (m·v‐1) methionine/drinking water for 3 months, then mice were divided into five groups for the next 2 month: the control mice, the 1% methionine group, the 1% methionine+P‐II group (treated orally with P‐II low dose:10 mg/kg, or high dose:60 mg/kg) or PBS every day, the 1% methionine+P‐II treatment (high dose:60 mg/kg)+EX527 group, which 1.2 mM EX527 was diluted in phosphate‐buffered saline (5 μl) was administered intravenously at 2 hours before P‐II treatment. (A‐C) The mRNA levels of IL‐6(A), CXCL15 (human IL‐8 homologous) (B) and TNF‐α (C) in the thoracic arteries were assessed by real‐time PCR; (D‐F).The plasma concentrations of IL‐6(D), CXCL15(E) and TNF‐α (F) were determined by ELISA. The data are presented as the mean ± SD (n = 10 in each group). *P < 0.05 as compared to control group mice, ^#^ *P* < 0.05 as compared to hyperhomocysteinaemic mice. ^&^ *P* < 0.05 as compared to the 1% methionine + P‐II treatment (high dose: 60 mg/kg) group mice

HHcy increased the expression of CXCL15 (human IL‐8 homologous), TNF‐α and IL‐6 in the thoracic arteries of HHcy mice. Similarly, plasma levels of HHcy‐associated inflammatory cytokines were also elevated. These changes in inflammatory cytokines were reversed after P‐II treatment (Figure 3A‐F).

### 3.3 Expression of LOX‐1, SIRT1 in control and hyperhomocysteinaemic mice

**Figure 4:**
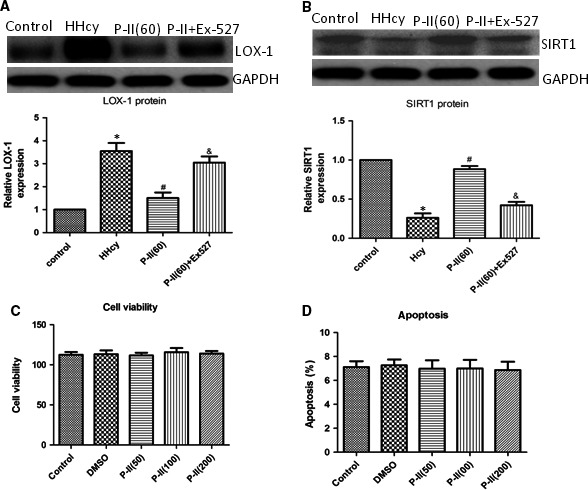
The effect of P‐II on vascular LOX‐1 and SIRT1 expression in hyperhomocysteinaemic mice. The control mice (Control) had free access to water, and the hyperhomocysteinaemic mice (HHcy) were treated with 2% (m·v‐1) methionine/drinking water for 3 months, then mice were divided into four groups for the next 2 month: the control mice, the 1% methionine group, the 1% methionine+P‐II group (treated orally with high dose:60 mg/kg), the 1% methionine+P‐II treatment (high dose:60 mg/kg)+EX527 group, which 1.2 mM EX527 was diluted in phosphate‐buffered saline (5 μl) was administered intravenously at 2 hours before P‐II treatment. (A) Vascular LOX‐1 expression was determined by Western blot analysis; (B) Vascular SIRT1 expression was determined by Western blot analysis. (C, D) HUVECs were treated with different concentrations of P‐II(50 μg/ml, 100 μg/ml, 200 μg/ml) for 48 hours, then cell viability (C) and cell apoptosis were measured (D). The data are presented as the mean ± SD (n = 10 in each group). *P < 0.05 as compared to control group mice, ^#^ *P* < 0.05 as compared to hyperhomocysteinaemic mice. ^&^ *P* < 0.05 as compared to the 1% methionine+P‐II treatment (high dose: 60 mg/kg) group mice

Compared with control animals, the LOX‐1 protein expression increased and SIRT1 expression decreased markedly in the HHcy group (Figure 4A,B), however, P‐II treatment markedly increased SIRT1 and decreased LOX‐1 expression compared to the HHcy group (*P *<* *0.05) (Figure 4A,B). Moreover, EX527 partly abolished the P‐II‐induced protection against HHcy‐induced SIRT1 inhibition (Figure 4B), NADPH oxidase activation (Figure 2A), LOX‐1 up‐regulation (Figure 4A), and the increase of pro‐inflammatory cytokines production (Figure 3A‐F).

### 3.4 Toxicity of picroside II to HUVECs

As shown in Figure 4C,D, cell viability and cell apoptosis were not affected by treatment of HUVECs with 50 μg/mL, 100 μg/mL, 200 μg/mL of P‐II for 48 hours.

### 3.5 Picroside II Inhibits ROS formation and NADPH oxidase activation in HUVECs

**Figure 5:**
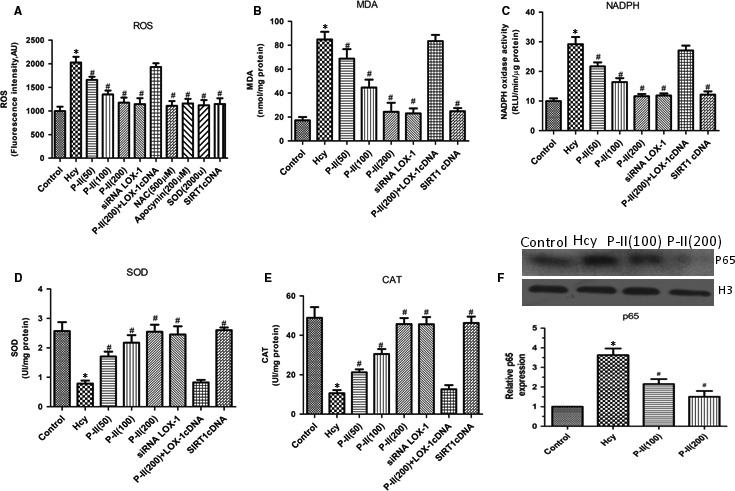
Effects of P‐II on oxidative stress in HUVECs induced by Hcy. HUVECs were stimulated with Hcy alone or in combination with different concentrations of P‐II, or siRNA LOX‐1, OR SIRT1 cDNA. (A). Hcy significantly increase ROS production after treatment for 24 hours, but pretreated with P‐II or siRNA LOX‐1 or SIRT1 cDNA for 24 hours, Hcy‐induced ROS production were decreased markedly; (B‐E). HUVECs that were treated with Hcy for 24 hours demonstrated a significant increase in MDA levels (B) and NADPH oxidase activity (C), and concomitant decrease in the activity of SOD (D) and CAT (E), P‐II or siRNA LOX‐1 or SIRT1 cDNA prevent Hcy‐induced lipid peroxidation, reduced NADPH oxidase activation and increased the activity of antioxidant enzymes in HUVECs (B‐E). Overexpressing of LOX‐1 (LOX‐1cDNA) partly abolished the P‐II‐induced protection against Hcy‐caused NADPH oxidase activation (Figure 6A‐E). (F) NF‐κBp65 was determined with nuclear protein from endothelial tissues. The data are shown as the mean ±SD of six separate experiments. *P < 0.05 as compared to control group cells, ^#^ *P* < 0.05 as compared to Hcy‐treated cells

HHcy significantly increased ROS and MDA levels and NAPDH oxidase activity, whereas preincubation with P‐II reduced the HHcy‐induced NADPH oxidase activation and ROS formation in a concentration‐dependent manner (Figure 5A‐C). As shown in (Figure 5D,E), addition of Hcy resulted in a marked decrease in SOD and CAT activity, however, P‐II at 50, 100 and 200 μg/mL, NAC and Apocynin significantly restored the suppression of SOD and CAT activity induced by HHcy.

### 3.6 Picroside II mediated the homocysteine induced endothelial inflammation in HUVECs

**Figure 6:**
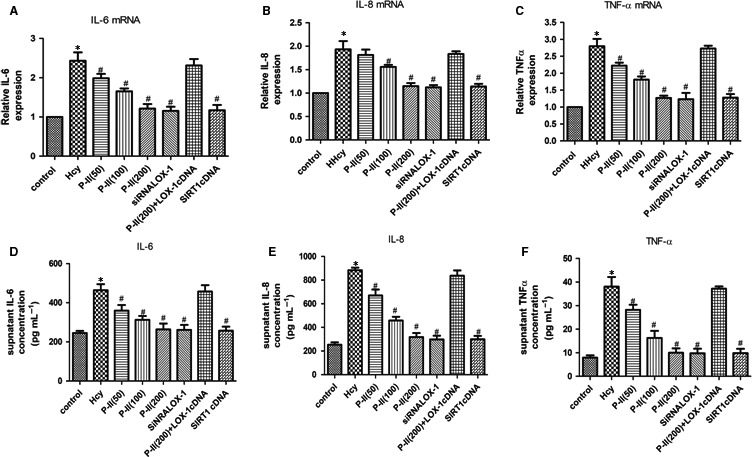
P‐II attenuated Hcy‐induced endothelial inflammation in HUVECs. HUVECs were incubated for 24 hours or 48 hours in the absence or presence of P‐II or siRNA LOX‐1 or SIRT1 cDNA, followed by the addition of 100 μmol Hcy for next 24 hours. (A‐F). P‐II inhibits Hcy‐induced up‐regulation of inflammatory cytokines. The mRNA levels and supernatant levels of IL‐6, IL‐8 and TNF‐α were determined separately by Real‐time PCR (A, B, C) and ELISA (E,F,G). The data are shown as the mean ± SD of six independent experiments. *P < 0.05 as compared to control group cells, ^#^ *P* < 0.05 as compared to Hcy‐treated cells

A shown in (Figure 5F), HHcy induce the activation of NF‐κB, as indicated by nuclear translocation and DNA binding of its p65 subunit, which was inhibited by P‐II in a concentration dependent manner. Furthermore, pretreatment with P‐II reversed the up‐regulation of IL‐8, IL‐6 and TNF‐α induced by HHcy (Figure 6A‐F).

### 3.7 Picroside II reduces HHcy‐induced endothelial apoptosis in HUVECs

**Figure 7:**
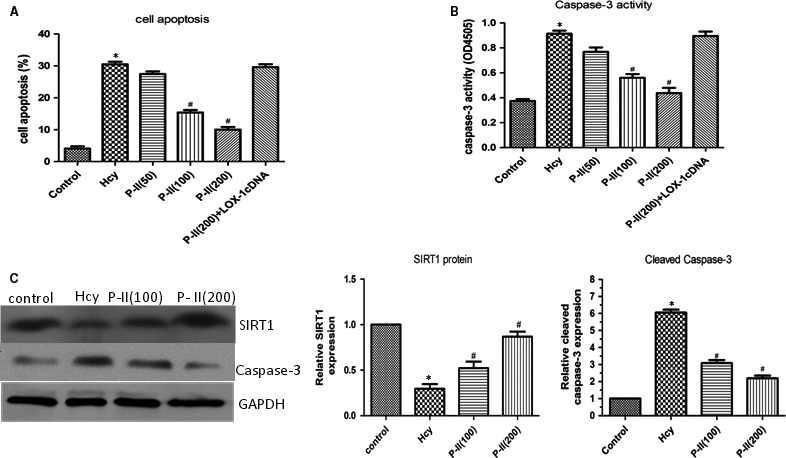
The effect of P‐II on Hcy‐ indued Caspase‐3 activation and endothelial cell apoptosis. HUVECs were stimulated with Hcy alone or in combination with different concentrations of picroside II, or siRNA LOX‐1, OR SIRT1 cDNA. (A). Annexin V‐FITC assay was described to determine the apoptotic cells. Hcy significantly increased cell apoptosis after treatment for 24 hours, but pretreated with P‐II or siRNA LOX‐1 or SIRT1 cDNA for 24 hours or 48 hours, Hcy‐induced cell apoptosis were decreased markedly; (B). Using caspase‐3 assay kit, Caspase‐3 activity was studied. HUVECs treated with Hcy for 24 hours demonstrated a significant increase in Caspase‐3 activity (B), P‐II or siRNA LOX‐1 or SIRT1 cDNA prevent Hcy‐induced Caspase‐3 activation; (C) Hcy significantly increase cleaved Caspase‐3 expression, meanwhile decrease SIRT1expression, but pretreated with P‐II, Hcy‐induced Caspase‐3 expression were decreased and SIRT1 expression were increased markedly in HUVECs (C)

HHcy resulted in significant increase in HUVECs apoptosis. When cells were incubated with P‐II, the apoptotic process was dramatically down‐regulated in a concentration‐dependent manner (Figure 7A). The Caspase‐3 activity displayed a similar change after adding P‐II into the culture media of HUVECs. Western blotting showed the activity of Caspase‐3 and the cleaved Caspase‐3 levels were obviously elevated after HHcy. When the cells were pretreated with increasing concentrations of P‐II, the Caspase‐3 activity and the cleaved Caspase‐3 levels were gradually decreased (Figure 7B,C).

### 3.8 The LOX‐1 receptor and SIRT1 participate in P‐II ‐involved cell protection

**Figure 8:**
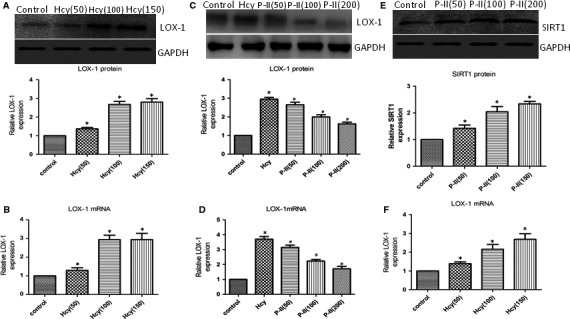
LOX‐1 expression in HUVECs induced by Hcy. (A‐B). HUVECs were incubated with 50‐150 μmol Hcy, the LOX‐1 expresion in HUVECs was assessed by Western blot (A) and Real‐time PCR(B); (C, D) HUVECs pretreated with different of P‐II for 24 hours, next with 100 μmol Hcy, the expression of LOX‐1 in HUVECs was assessed by Western blot (C) and Real‐time PCR (D). (E, F) SIRT1 expression in HUVECs induced by different concentrations of P‐II. HUVECs were incubated with 50‐200 μmol P‐II, the SIRT1 expression in HUVECs were assessed by Western blot (A) and Real‐time PCR (B).The data are shown as the mean ± SD of six independent experiments. *P <* *0.05 as compared to control group cells, ^#^ *P *< 0.05 as compared to Hcy‐treated cells

**Figure 9:**
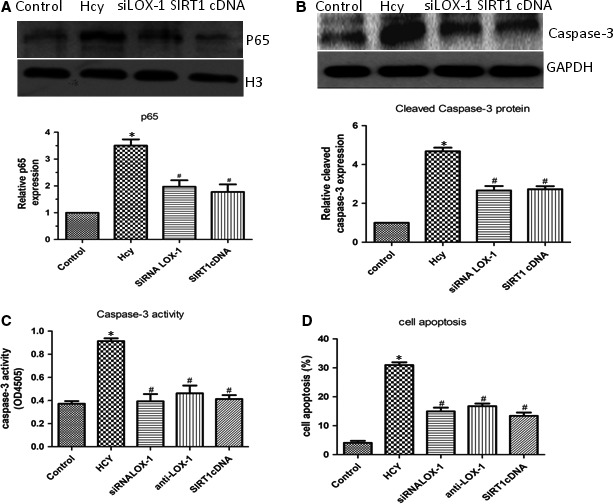
SIRT1/LOX‐1 axis regulates Hcy‐induced NF‐κBp65 activation and cell apoptosis. (A‐B) HUVECs were incubated with siRNA LOX‐1 or SIRT1cDNA, the NF‐κBp65 activation (A) and expression of Caspase‐3 (B) in HUVECs was determined by Western blot; (C, D) HUVECs were incubated with siRNA LOX‐1 or SIRT1cDNA, the Caspase‐3 activity in HUVECs and cell apoptosis were assessed. The data are shown as the mean ± SD of six independent experiments. *P < 0.05 as compared to control group cells, ^#^ *P* < 0.05 as compared to Hcy‐treated cells

Treatment with 100 and 150 μM Hcy, The mRNA and protein levels of LOX‐1 were dramatically increased in human endothelial cells (Figure 8A,B), whereas siRNA‐LOX‐1 reversed the NF‐κB activation (Figure 9A) and IL‐6, IL‐8 and TNF‐α production induced by HHcy (Figure 6A‐F). In addition, HHcy‐induced ROS formation (Figure 5A) and cell apoptosis (Figure 7A) were abrogated by pretreatment with siRNA LOX‐1. Treatment with P‐II decreased concentration–dependently LOX‐1 expression in endothelial cells (Figure 8C,D). Overexpressing of LOX‐1 (LOX‐1cDNA), however, partly abolished the P‐II‐induced protection against HHcy‐ induced NADPH oxidase activation (Figure 5A‐E) as well as pro‐inflammatory cytokines production (Figure 6A‐F) and cell apoptosis (Figure 7A,B).

**Figure 10:**
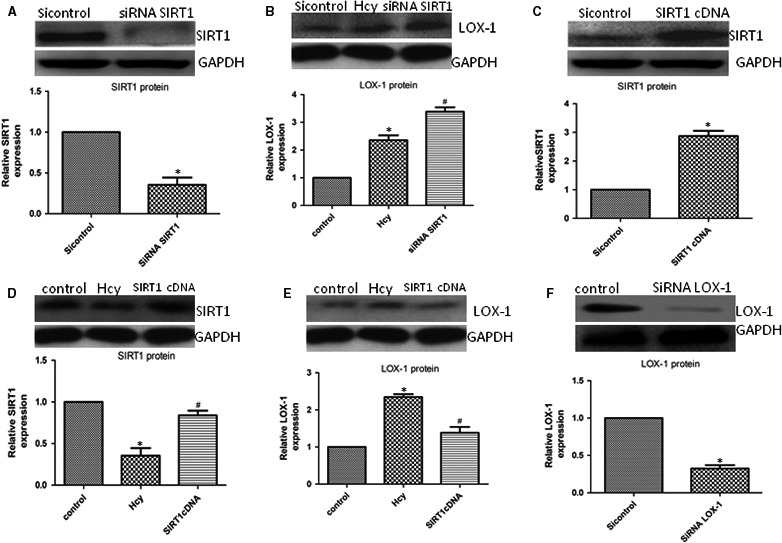
LOX‐1 expression by the inhibition of the SIRT1 axis. (A, B) HUVECs were incubated with siRNA SIRT1, SIRT1(A) or LOX‐1(B) expression in HUVECs were assessed using Western blot; (C) HUVECs were incubated with SIRT1 cDNA, the SIRT1 expression in HUVECs were determined by Western blot; (D, E) HUVECs were transfected with SIRT1 cDNA before exposure to Hcy, the expression of SIRT1(D)or LOX‐1(E) in HUVECs were assessed by Western blot. (F) HUVECs were incubated with siRNA LOX‐1, the LOX‐1 expression in HUVECs were assessed using Western blot. The data are shown as the mean ± SD of six independent experiments. *P < 0.05 as compared to control group cells, ^#^ *P* < 0.05 as compared to Hcy‐treated cells

Furthermore, overexpressing of SIRT1 reversed NF‐κB activation (Figure 9A), ROS formation (Figure 5A‐C), IL‐6, IL‐8, TNF‐α production (Figure 6A‐F) and cell apoptosis induced by HHcy (Figure 9B‐D). Interestingly, blockade of SIRT1 with siRNA SIRT1 decreased SIRT1 (Figure 10A) and increased LOX‐1(Figure 10B), whereas overexpressing of SIRT1 with SIRT1cDNA increased SIRT1 (Figure 10C,D) and decreased LOX‐1 (Figure 10E).

## 4 DISCUSSION

The results from our study showed that P‐II, the main active ingredient in the root department of Chinese medicine Picrorhiza scrophulariiflora, could reduce HHcy‐induced endothelial oxidase stress, inflammation and cell apoptosis in vitro and in vivo.

**Figure 11:**
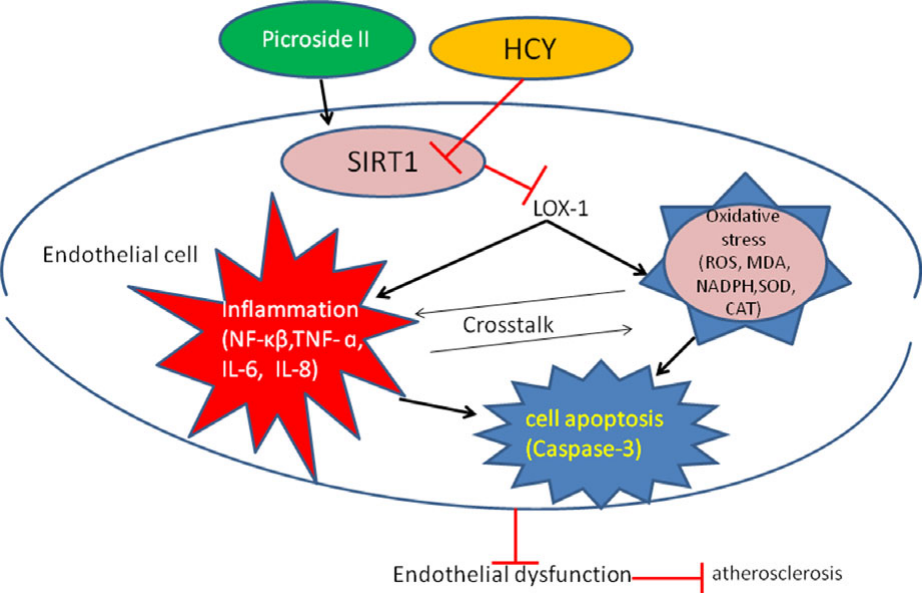
Schematic illustration of the possible targeting and signaling by which P‐II restore endothelial dysfunction induced by HHCY

Emerging evidence suggests that HHcy accelerates atherosclerotic process as a result of increased oxidative stress.^22^ Intracellular ROS levels are regulated by the balance between ROS formation and antioxidant enzymes.^23^ In endothelial cells, NADPH oxidase is identified as a major source of oxidative stress.^24^ Consistent with previous reports,^8^, ^25^ the present study demonstrated that HHcy increased NADPH oxidase activation, lipid peroxidation and MDA elevation, and markedly decreased the activity of antioxidant enzymes, CAT and SOD. Our results also shown a marked reduction in HHcy‐induced elevation of ROS, MDA and NADPH oxidase activation after pretreatment with P‐II. In addition, P‐II reversed HHcy‐induced reduction in SOD and catalase activities in vitro and in vivo. These findings support a notion that P‐II restores endothelial function by normalization of ROS and antioxidant defenses (Figure 11).

ROS can activate NF‐*κ*B and enable nuclear translocation and subsequent regulation of proinflammatory molecules, including cytokines, chemokines and adhesion molecules. Once NF‐*κ*B is activated, it translocates from the cell cytosol to the nucleus, binds to specific DNA sequences, and initiates transcription. These changes could alter gene expression, and induces the caspase‐3 and final common effector proteases activation, eventually leading to apoptosis.^24^ In this study, we found that ROS generation in HUVECs occurred within 20 minutes (data not shown), and within 4 hours of the incubation of Hcy, NF‐*κ*B was activated. P‐ II inhibited NF‐*κ*B activation and repressed HHcy‐induced IL‐6, IL‐8, TNF‐a expression. We speculated that the protective effects of P‐II against HHcy‐induced endothelial damage might be through inhibiting intracellular ROS formation and proinflammatory cytokines production. Taken together, these observations strongly indicate that P‐II elicits antioxidative and anti‐inflammatory effects.

LOX‐1, primary oxLDL receptor on endothelial cells, plays an important role in the pathogenesis of atherosclerosis.^26^, ^27^, ^28^ OxLDL, angiotensin II or other pro‐inflammatory events have been reported to activate LOX‐1 expression.^29^ Recently, several studies have reported a link between LOX‐1 and HHcy‐induced endothelial dysfunction.^8^, ^25^, ^30^ LOX‐1 up‐regulation by HHcy stimulates endothelial proinflammatory cytokines production and generation of superoxide radicals, consequently leading to cell apoptosis.^8^ In the present study, we found that HHcy increased LOX‐1 mRNA and protein expression, whereas blockade of LOX‐1 with siRNA LOX‐1 resulted in marked reduction in ROS formation, NF‐*κ*B activation, proinflammatory molecules expression and cell apoptosis. This suggests that HHcy binds to LOX‐1 which leads to consequent changes. Additionally, P‐II decreased LOX‐1 expression, inhibited NF‐*κ*B activation, repressed Hcy‐induced IL‐8, TNF‐a and IL‐6 production, decreased caspases‐3 activity and cell apoptosis, by comparison, overexpression of LOX‐1 attenuated P‐II protection. From these, we speculated that the protective effects of P‐II on HHcy‐induced endothelial injury may be through blockading the LOX‐1.

SIRT1, an important anti‐atherosclerosis molecule, protects the cardiovascular system from degeneration and oxidative injuries.^31^, ^32^ Previous reports have suggested that SIRT1 activation is an effective approach to manage HHcy‐induced oxidative stress,^33^, ^34^ and SIRT1 decreased the LOX‐1 expression level and NF‐κB activation.^8^, ^25^ This statement is similar to our findings in the present study, as shown in Figures 4B and 7C, Hcy decreased SIRT1 expression significantly. However, blockade of SIRT1 with Ex527(in vivo) or siRNASIRT1(in vitro), LOX‐1 expression increased markedly, subsequently induced ROS production, NF‐*κ*B activation, proinflammatory molecules expression and cell apoptosis, by comparison, overexpression of SIRT1 decreased LOX‐1 expression markedly, which suggested that the binding of SIRT1 to LOX‐1, and the consequent changes. In addition, our study showed that P‐II increased SIRT1 expression, decreased LOX‐1 repression, inhibited NF‐*κ*B activation and ROS generation, repressed the Hcy‐induced IL‐6, IL‐8 and TNF‐a expression, finally decreased caspases‐3 activity and cell apoptosis, by comparison, blockade of SIRT1 with SIRT1 inhibitor, EX527, in vivo and SIRT1 siRNA in vitro, increased LOX‐1expression and attenuated P‐II protection. The data from our study demonstrated that P‐II attenuated the up‐regulation of HHcy‐induced LOX‐1 via activation of the ubiquitination of SIRT1. An overview of the analyzed pathways and results is given in Figure 11.

In summary, the results from our study indicated that P‐II prevented HHcy‐induced LOX‐1‐mediated HUVECs injury, probably with its anti‐oxidative and anti‐inflammatory effects.

## CONFLICT OF INTEREST

The authors have declared no conflict of interest.
